# HPLC-based metabolomics of *Dendrobium officinale* revealing its antioxidant ability

**DOI:** 10.3389/fpls.2023.1060242

**Published:** 2023-01-24

**Authors:** Zhengfei Luo, Lian Liu, Qiong Nie, Mingjin Huang, Chunlii Luo, Yedong Sun, Yongyan Ma, Jianxin Yu, Fuqiang Du

**Affiliations:** ^1^ College of Agriculture, Guizhou University, Guiyang, China; ^2^ Anlong County Xicheng Xiushu Agriculture and Forestry Co., Ltd, Anlong, China; ^3^ GuiZhou Warmen Pharmaceutical Co., Ltd, Guiyang, China

**Keywords:** Dendrobium officinale, metabolic profiling, differential metabolites, flavonoid, antioxidant activity

## Abstract

*Dendrobium officinale* is an orchid with medicinal and nutritional properties that has received increasing attention because of its health benefits; however, there is limited information about the metabolic basis of these properties. In this report, secondary metabolites and the antioxidant activity of *D. officinale* stem samples from three provenances were analyzed, using a UHPLC-QqQ-MS/MS-based metabolomics approach. In total, 411 metabolites were identified including 8 categories such as flavonoids and phenolic acids, 136 of which were differential metabolites. These differentially accumulated metabolites (DAMs) were mainly enriched in secondary metabolic pathways such as flavone, flavonol, tropane, piperidine, pyridine, isoquinoline alkaloid biosynthesis and tyrosine metabolism. The metabolomic profiling suggested that the quantity and content of flavonoid compounds accounted for the highest proportion of total metabolites. Hierarchical cluster analysis (HCA) showed that the marker metabolites of *D. officinale* from the three provenances were mainly flavonoids, alkaloids and phenolic acids. Correlation analysis identified that 48 differential metabolites showed a significant positive correlation with antioxidant capacity (r ³ 0.8 and p < 0.0092), and flavonoids were the main factors affecting the different antioxidant activities. It is worth noting that quercetin-3-O-sophoroside-7-O-rhamnoside and dihydropinosylvin methyl ether might be the main compounds causing the differences in antioxidant capacity of Yunnan provenance (YN), Zhejiang provenance (ZJ), and Guizhou provenance (GZ). These finding provides valuable information for screening varieties, quality control and product development of *D. officinale*.

## 1 Introduction

*Dendrobium officinale* Kimura & Migo, a perennial herb and belongs to the genus *Dendrobium* in the family *Orchidaceae* is a well-known perennial herb used as a medicinal and food homologous product, has been utilized for the treatment of yin-deficiency diseases for decades. *D. officinale* is rich in alkaloids, polysaccharides, flavonoids, terpenoids, stilbenes, phenols and lignins (Lam et al., 2015; Zhang et al., 2018; Cai and Peng, 2021), with potential pharmacological activity against cataracts (Huang et al., 2019), acts as a neuroprotective (Liu et al., 2020), controls diabetes, and displays antioxidant (Hostetler et al., 2017), antibacterial, immune regulatory (Xie et al., 2018; Sun et al., 2020; Xi et al., 2020), antitumor and antimutation effects (Li et al., 2019; He et al., 2020). In traditional Chinese medicine, the fresh or dry stem of *D. officinale* has been linked to generating fluid and benefiting the stomach, nourishing yin and clearing heat, protecting the liver and eyesight, relieving coughing and moistening the lungs (Meng et al., 2017; Yue et al., 2020), and famously known as one of the nine immortal herbs (Zhang et al., 2016).

Wild *D. officinale* grows mainly on damp trunks or limestone, and mainly distributed in tropical and subtropical areas between 15° 31’ north latitude and 25° 12’ south latitude, e.g., Myanmar, Vietnam and China’s Anhui, Zhejiang, Yunnan and Guizhou Provinces (Shiau et al., 2005; Pan et al., 2022).Wild *D. officinale* is scarce because it requires a peculiar growth environment and takes a long time to grow. Moreover, wild *D. officinale* has been exploited excessively due to the large demand from the market (Tang et al., 2017). In order to satisfy market demand, researchers tried to plant *D. officinale* by means of artificial cultivation (Si et al., 2013; Fan et al., 2015). Recently, the artificial planting technology of *D. officinale* has made breakthroughs, with the production quality increasing (Zeng et al., 2012; Mala et al., 2017). The cultivation method of *D. officinale* mainly include imitating wild cultivation (attached to trees and stones) and greenhouse cultivation. The contents of *Dendrobium* polysaccharides, polyphenols and flavonoids cultivated with imitation wild cultivation under a forest are higher than those in greenhouse cultivation (Si et al., 2013). However, the harvest of *D. officinale* using greenhouse cultivation is higher than that achieved by imitation wild cultivation (Huang et al., 2022). Artificial cultivation of *D. officinale* has expanded from the traditional Zhejiang and Yunnan Provinces to Guangxi, Guangdong, Anhui, Hunan and Guizhou Provinces. The forest coverage rate in Guizhou Province is as much as 60%, and planting *D. officinale*-attached trees or cultivating this orchid in three-dimensional planting under a forest has significant economic and ecological advantages. In 2020, the planting area of *D. officinale* in Guizhou was 4,522.26 ha, the largest of the country’s original ecological cultivation area. However, most of the *D. officinale* cultivated in Guizhou Province came from Zhejiang and Yunnan provenance, and the native provenances are less cultivated.

Chinese herbal medicine pays particular attention to authenticity. Specific provenances and climatic conditions have a specific effect on the growth and effect of medicinal herbs. There are obvious differences between the metabolites of *Dendrobium* from different origins, and the epiphytic cultivation mode of living trees under a forest can improve the biosynthesis levels of flavonoids and flavonoid compounds (Li et al., 2022; Fan et al., 2022). Several studies reported a close relationship between *D. officinale* from different regions and its physiological activities, especially the antioxidant activity (Xu et al., 2013; Zhang et al., 2019). Plant secondary metabolites are a arise from the interaction between plants and biological and abiotic factors during evolution. The active ingredients of Chinese medicinal herb are mostly the secondary metabolites, and the evaluation of quality and effectiveness of medicinal materials are based on the types and content of secondary metabolism. Metabolomics is a new omics technology after genomics and proteomics (Monteiro et al., 2013). Metabolomics have been widely used in the analysis of herbal composition (Li et al., 2022; Gao et al., 2022) and the mechanism of efficacy based on high-throughput detect the content of metabolites in samples qualitatively, quantitatively and with high coverage (Wei et al., 2010).

Currently, the planting *D. officinale*-attached trees area under forests in Guizhou is nearly 3,335 ha. The seedling sources are mainly from Zhejiang, Yunnan and Guizhou. The products have sold well in China and abroad, but there are only a few reports on their yield and quality characteristics. Anlong County, Guizhou Province, is one of the origins of *D. officinale*, which has had the advantage of wild *Dendrobium* growth since ancient times. The objectives of the present study were to: 1) detected the components and contents of secondary metabolites of *D. officinale* from three provenances planted in Anlong County, 2) measured the antioxidant activity of *D. officinale in vitro*, to analyze the correlation between metabolites and antioxidant activity, 3) comprehensive analysis of the composition and quality of *D. officinale* can provide reference for the selection and cultivation of excellent varieties, and making better use of local provenance. Our overall aim was that used the morphology, metabolomics and HPLC technology to analyses the phenotype, secondary metabolite composition and antioxidant activity of fresh stems of *D. officinale* from three provenances planted in Anlong County, to understand the correlation between the different metabolites and antioxidant activities.

## 2 Materials and methods

### 2.1 Experimental materials and preparation of samples

**Figure 1:**
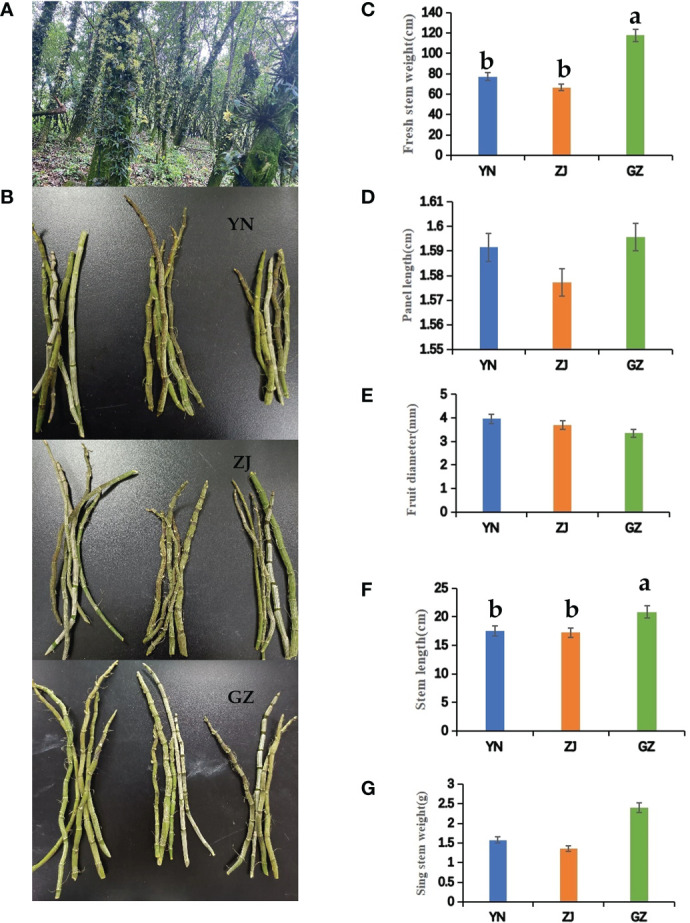
Agronomic traits of *Dendrobium officinale* from different provenances. **(A)** Attached to the tree planting. **(B)** Photographs showing *Dendrobium officinale* from different provenances, YN; ZJ; GZ. **(C)** fresh stem weight; **(D)** panel length; **(E)** fruit diameter; **(F)** stem length; **(G)** single stem weight. Different lowercase letters represent significant differences.

Three *D. officinale* from Yunnan provenance (YN), Zhejiang provenance (ZJ) and Guizhou Anlong native provenance (GZ) were cultivated in Anlong County Zhegui village, GZ Province, and it is cultivated by attaching trees (**Figure 1A**). YN and ZJ materials were introduced and cultivated from Guangnan County of Yunnan Province and Yandang Mountain of Zhejiang Province in March and August 2016, respectively. In August of the same year, Anlong native species (GZ) were planted in the nearby forest land, with an area of 6.67 ha each. The same management mode of wild imitating cultivation is adopted. In December 2021, the “5-point sampling method” was adopted to take 2–3-year-old stems of *D. officinale* as experimental samples. A total of 200 stems were collected from each variety for mixing, and the stem phenotypes of 49 *D. officinale* were randomly determined. All stems were sliced and evenly mixed, and three pieces were randomly taken as a sample. Three biological replicates were conducted. Nine samples were treated with liquid nitrogen and stored at –80°C for metabolome analysis. The remaining materials were dried, ground and passed through a No. 3 sieve for antioxidant analysis and flavonoid determination.

### 2.2 Determination of antioxidant activity

An alcohol extraction approach was used for the antioxidant analysis of *D. officinale*. Two grams of *D. officinale* powder was weighed into a 50 mL centrifuge tube. Fifty milliliters of 70% (v/v) ethanol were added, the extract ultrasonicated in a 50°C water bath for 1.5 h, centrifuged (5000 r/min, 2 min) and filtered, and the filtered residue was extracted by the same method for 1 h. The filtrate was combined and transferred to a 100 mL volumetric flask and 70% (v/v) ethanol added to a volume that yielded a 20 mg/mL sample, which was stored at –20°C until use. Three antioxidant methods, DPPH, ABTS and FRAP, were used to examine the antioxidant properties of samples using the instructions in each kit (A153-1-1, A015-2-1, A015-3-1, NanJing JianCheng Bioengineering Institute) and a microplate reader (Multiskan GO, Thermo Scientific, Massachusetts, USA).

### 2.3 Flavonoid content determination

#### 2.3.1 Chromatographic conditions

A Z0RBAX SB-C18 column (4.6 mm x 250 mm, 5 mm) was used. The gradient elution conditions were: 0 to 10 min, 10% to 11% solvent A, where solvent A was tetrahydrofuran: acetonitrile: methanol (10:22:5) and solvent B was 0.05% aqueous phosphate; 10–25 min, 11%–11.5% solvent A; 25–32 min, 11.5%–12% solvent A; 32–42 min, 2%–12.5% solvent A; 42–52 min, 12.5%–13.5% solvent A; 52–75 min, 13.5%–14% solvent A; and 75–85 min, 14%–20% solvent A. The flow rate was 1 mL min^–1^, the detection wavelength was 340 nm to determine the five flavonoid components (C1–C5), the column temperature was 30°C, and effects of inject ion volume was 10 µL.

#### 2.3.2 Preparation of control solutions

Reference materials vicenin-2, schaftoside, isoschaftoside, isoviolanthin and violanthin (C1–C5) were accurately weighed and methanol was added to dissolve each reference. A single component control stock solution was then mixed and shaken well. The appropriate amount of the abovementioned control stock solution was taken, and methanol was added to make a mixed control solution. The mixed control solution prepared above was further diluted into five control solutions of different mass concentrations. The injection mass concentration *X* (mg L^–1^) and the chromatographic peak area *Y* were taken to obtain the regression equation (C1 vicenin-2: y = 5.8944x + 9.9518, R^2^ = 0.9985; C2 schaftoside: y = 25.762x – 23.996, R^2^ = 0.9945; C3 isoschaftoside: y = 9.2539x + 10.91, R^2^ = 0.9959; C4 isoviolanthin: y = 9.7296x + 59.31, R^2^ = 0.9968; C5 violanthin: y = 10.567x + 18.353, R^2^ = 0.998).

#### 2.3.3 Preparation of sample solutions for testing

Dendrobium powder (through a no. 3 sieve) was taken and dried to a constant weight of ~4 g. The precise weight was measured, and 200 mL 80% (v/v) methanol was added. The sample was heat refluxed for 2 h and cooled to room temperature. The sample was filtered, the filter residue was washed with an appropriate amount of 80% (v/v) methanol, the filtrates combined, the solvent evaporated, and 80% (v/v) methanol was added to dissolve the material. Ten milliliters were added to a measuring bottle, and 80% (v/v) methanol was added. The sample was shaken well and filtered with a 0.45 μm microporous filter membrane to obtain the desired product.

#### 2.3.4 Determination of flavonoids

The content of flavonoid carbonoside was determined by HPLC (Agilent 1260), refer to the literature (Tang Y et al., 2021). In brief, the precision absorption control solution and the test solution were 10 μL. Sample injection and analysis were carried out according to instructions. Flavonoid amounts were determined using the standard curve equations (2.3.2).

### 2.4 Secondary metabolites detection by UPLC-MS/MS

#### 2.4.1 Sample preparation and extraction

Biological samples were lyophilized using a vacuum freeze-dryer (Scientz-100F) and crushed using a mixer mill (MM 400, Retsch) with a zirconia bead for 1.5 min at 30 Hz. The lyophilized powder (100 mg) was dissolved in 1.2 mL, 70% methanol, mixed for 30 s every 30 min six times and placed at 4°C overnight. Following centrifugation at 12000 rpm for 10 min, the extracts were filtered (SCAA-104, 0.22 μm pore size; ANPEL, Shanghai, China) and analyzed by UPLC-MS/MS.

#### 2.4.2 UPLC conditions, ESI-Q TRAP-MS/MS and qualitative and quantitative analysis of metabolites

UPLC conditions and ESI-Q TRAP-MS/MS parameters reported in reference [Tang et al., 2021] were used. Based on the local metabolic database, the metabolites of the sample were qualitatively quantified by MS. The multi-reaction monitoring mode (MRM) and metabolite detection multimodal diagram were used to identify the substances detected in the sample. The characteristic ions of each substance were screened by the triple four-stage rod, and the signal strength (CPS) of the characteristic ions was obtained using the detector. The sample data were analyzed with MultiQuant, and the integration and correction of the MS peaks were carried out. The peak area of each MS peak represents the relative content of the corresponding substance, and all MS peak integration data was exported for preservation.

#### 2.4.3 Treatment of data

Logarithmic transformations were performed on the abundant original metabolites to normalize the data and achieve homogeneity of variances. Principal component analysis (PCA) and quadrature partial least squares discriminant analysis (OPLS-DA) were performed using R (www.r-project.org/). Hotelling’s T-squared ellipse method was used to determine the sample repeatability and confidence interval. Heat maps were drawn using the R ComplexHeatmap package, and Pearson correlation coefficients were calculated using the built-in cor function of R. Metabolites satisfying the following two criteria were selected as differential metabolites between two prov (YN vs. ZJ, YN vs. GZ, ZJ vs. GZ): (i) high confidence (VIP ≥ 1) in pairwise comparisons; (ii) a minimum of a two-fold change or a maximum of 0.5-fold change (fold change ≥ 2 and fold change ≤ 0.5). Differential metabolites were annotated and classified using the Kyoto Encyclopedia of Genes and Genomes (KEGG) database (http://www.kegg.jp/kegg/pathway.html). Significantly enriched pathways in which the metabolites in a module were involved were compared with the background and defined by both a hypergeometric test and a threshold *p*-value < 0.05.

Microsoft Office Excel 2019 was used for the initial collation of data, and analysis of variance (ANOVA) was used with PASS 27.0.1. The results were expressed as mean ± standard deviation (SD). The significance of the sample-to-sample difference was determined using one-way ANOVA and Duncan’s Multiple Range Test with a significance level of 0.05. For secondary metabolome data, significantly regulated metabolites between groups were determined by Variable Importance in Projection (VIP) ≥ 1 and absolute log2FC (fold change) ≥ 1. VIP values were extracted from the OPLS-DA results, which also contained score plots and permutation plots and were generated using the R package MetaboAnalystR. The data were log-transformed (log2) and mean-centered before OPLS-DA. To avoid overfitting, a permutation test (200 permutations) was performed. The functions of differentially accumulated metabolites (DAMs) were annotated based on the KEGG compound database to determine the metabolic pathways.

## 3 Results

### 3.1 Stem morphology of *D. officinale* from different provenances

The shape and size of fresh stems of *D. officinale* were the main factors determining the yield. The stem length of *D. officinale* from different provenances varies significantly. The stem lengths of samples taken from GZ are significantly longer than those from YN and ZJ, and the internode length was the longest among samples taken from the three provenances. The phenotype of the internode and stem lengths was the same, with YN samples measured as the second. GZ fresh and single stem weights were heavier than the corresponding weights from YN and ZJ samples. In contrast, the stem diameter of GZ samples was smaller than ZJ and YN but not significant (**Figures 1B–G**).

### 3.2 Antioxidant activity analysis of *D. officinale* from different provenances

DPPH radical scavenging activities, radical cation ABTS+ scavenging activities and ferric reducing antioxidant power (FRAP) are listed in (**Table 1**). The antioxidant capacity of the two extraction methods (alcohol extraction and water extraction) showed the same trend in *D. officinale* from three different Provenances, indicating the reliability of the antioxidant activity value. The DPPH value of GZ was significantly higher than that of YN. The ABTS radical cation scavenging activities of *D. officinale* from the three provenances ranged between 0.2126 and 0.7758 mmol/L. The ABTS values from YN were significantly lower when compared with fractions from GZ and ZJ, and the GZ fractions displayed the highest ABTS values among the fractions from the three provenances. The FRAP values of GZ fractions were the highest, followed by fractions from ZJ. A comparison of all DPPH, ABTS and FRAP values among the stem samples taken from the three provenances showed that GZ fractions had the highest antioxidant activities and YN fractions had the lowest.

**Table 1:** Antioxidant activities of *D. officinale* from different provenances.

Materials	AE			WE		
	(DPPH μg/mL)	(ABTS mmol/L)	(FRAP mmol/L)	(DPPH μg/mL)	(ABTS mmol/L)	(FRAP mmol/L)
GZ	2737.75 ± 16.24 a	0.7758 ± 0.04 a	4.7239 ± 0.34 a	2426.93 ± 17.86 a	0.6414 ± 0.01 a	4.3851 ± 0.05 a
ZJ	2711.95 ± 0.78 ab	0.6193 ± 0.01 b	3.9651 ± 0.21 b	2279.65 ± 4.29 b	0.4900 ± 0.01 b	3.5519 ± 0.01 b
YN	2703.06 ± 7.06 b	0.5184 ± 0.04 c	3.4909 ± 0.09 b	2108.06 ± 21.59 c	0.2126 ± 0.02 c	2.7547 ± 0.03 c

AE are alcohol extraction methods; WE are water extration methods. DPPH, 2,2-diphenyl-1-picrylhydrazyl radical scavenging ability; ABTS, 2,2’-Azinobis- (3-ethylbenzthiazoline-6-sulphonate; FRAP, ferric reducing antioxidant power. If the value of ABTS and FRAP from the extract is 1mM, the antioxidant capacity is equivalent to 1mM Trolox. Different letters followed the numbers indicate statistically significant differences (p < 0.05). Values are expressed as the mean ± SD, on a dry basis.

### 3.3 Flavonoid carbonoside components and contents analysis of *D. officinale* from different provenances

The content of flavonoid carbonoside in *D. officinale* fractions from the three provenances is shown in **Table 2**. The contents of vicenin-2 (C1), isoschaftoside (C3) and isoviolanthin (C4) in *D. officinale* fractions from GZ were significantly greater than those from ZJ and YN, and the contents of vicenin-2 (C1) and isoschaftoside (C3) in *D. officinale* fractions from ZJ were significantly higher than those from YN. However, there was no significant difference in the content of C4 from *D. officinale* fractions derived from ZJ and YN. Schaftoside (C2) and violanthin (C5) were not detected in *D. officinale* fractions from GZ, and violanthin (C5) was not detected in *D. officinale* fractions from ZJ. The total flavonoid carbonoside was higher in GZ than in ZJ and YN, indicating that the content of flavonoid carbonoside and antioxidant capacity of *D. officinale* from different provenances had the same trend.

**Table 2:** Total flavonoid carbonoside and reference substance content of *D. officinale* from different provenances(C1-C5).

Materials	C1	C2	C3	C4	C5	Total flavonoid carbonoside
	μg/g	μg/g	μg/g	μg/g	μg/g	μg/g
GZ	68.44 ± 0.33a	0	51.31 ± 0.22a	29.48 ± 0.36a	0	149.22 ± 0.67a
ZJ	54.38 ± 0.08b	6.31 ± 0.09	43.31 ± 0.33b	12.66 ± 0.22b	0	116.66 ± 1.02b
YN	41.37 ± 0.63c	6.69 ± 0.10	40.76 ± 0.06c	12.91 ± 0.21b	14.98 ± 1.13	116.71 ± 0.36b

C1: vicenin-2; C2: schaftoside; C3: isoschaftoside; C4: isoviolanthin; C5: violanthin. Different letters followed the numbers indicate statistically significant differences (p < 0.05). Values are expressed as the mean ± SD, on a dry basis.

### 3.4 Qualitative metabolic profiling of *D. officinale* stems

UPLC-MS/MS was used to analyze differences in metabolic components among *D. officinale* stems taken from GZ, ZJ and YN provenances. Based on the local metabolite database (metware database), qualitative and quantitative MS analyses were conducted on the metabolites in the samples. Four hundred eleven metabolites were identified and divided into eight known first-order categories according to their structure. These metabolites included 143 flavonoids, 128 phenolic acids, 68 alkaloids, 28 lignans and coumarins, 12 quinones, 6 terpenoids, 3 tannins and 23 other metabolites (**Table 3**). The 143 flavonoid metabolites included 42 flavonols, 39 flavones, 33 flavonoid carbonosides, 15 flavanones, 7 chalcones, 5 flavanols and 2 flavanonols. The 68 alkaloids metabolites included 28 alkaloids, 17 phenolamine, 10 plumerane, 6 pyridine alkaloids, 5 piperidine alkaloids, 1 benzylphenylethylamine alkaloid and 1 sesquiterpene alkaloid.

**Table 3:** Number of differential metabolites in the leaves of *Dendrobium officinale* from different provenances.

Group Class	YN vs ZJ		YN vs GZ		ZJ vs GZ	
	Up	Down	Up	Down	Up	Down
Flavonoids	17	8	35	10	10	10
Alkaloids	16	0	10	2	2	4
Phenolic acids	13	2	9	3	8	10
Lignans and Coumarins	2	1	1	2	1	3
Tannins	0	0	1	1	0	1
Quinones	1	1	0	0	0	5
Terpenoids	2	0	0	0		
Others	3	2	5	1	4	0
Total	68		80		58	

### 3.5 Principal component analysis and orthogonal projections to latent structures-discriminant analysis for stems of *D. officinale* from the three provenances

**Figure 2:**
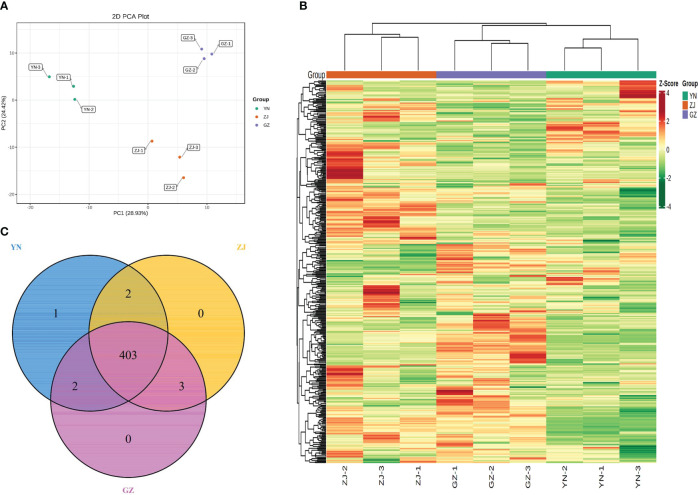
Overview of metabolites analysis detected in three *Dendrobium officinale* provenances. **(A)** Principal component analysis of the relative of metabolites for YN, ZJ and GZ: **(B)** Cluster heatmap of metabolite content in different samples. **(C)** Venn diagram of metabolite distribution in different materials.

The differences among the three *D. officinale* provenances were distinguishable and verified by 2D PCA (**Figure 2A**). In the PCA score plot, two principal components (PC1 and PC2) were extracted to be 28.93% and 24.42%, respectively. The results showed that the three *D. officinale* provenances were clearly separated, and three biological replicates of each variety were compactly gathered together, indicating that the experiment was reproducible and reliable. This comparison indicated significant differences between the three provenances, with all samples falling within the 95% confidence intervals. In addition, the cluster heatmap and class heatmap of the metabolites clearly showed the similarity of components among biological repeats and the difference of components among different species (**Figure 2B**). These results suggest that the provenance strongly influences the metabolite profiles of different *D. officinale*. Venn diagram analysis of the substances contained in the different species showed that 403 compounds were common to all three provenances, with only one specific metabolite (4-acetoxy-3-ethoxybenzaldehyde) found in *D. officinale* from the YN provenance and three metabolites (luteolin, 1-O-Feruloyl-β-D-glucose and 6-O-Feruloyl-β-D-glucose) were only present in extracts of ZJ and GZ. Two metabolites (2,4,6,6-Tetramethyl-3(6H)-pyridinone and vitexin-7-O-rutinoside) were only present in extracts of YN and ZJ, and two metabolites (fraxetin and 3-Hydroxy-4-methoxybenzoic acid) were only present in extracts of YN and GZ (**Figure 2C**).

**Figure 3:**
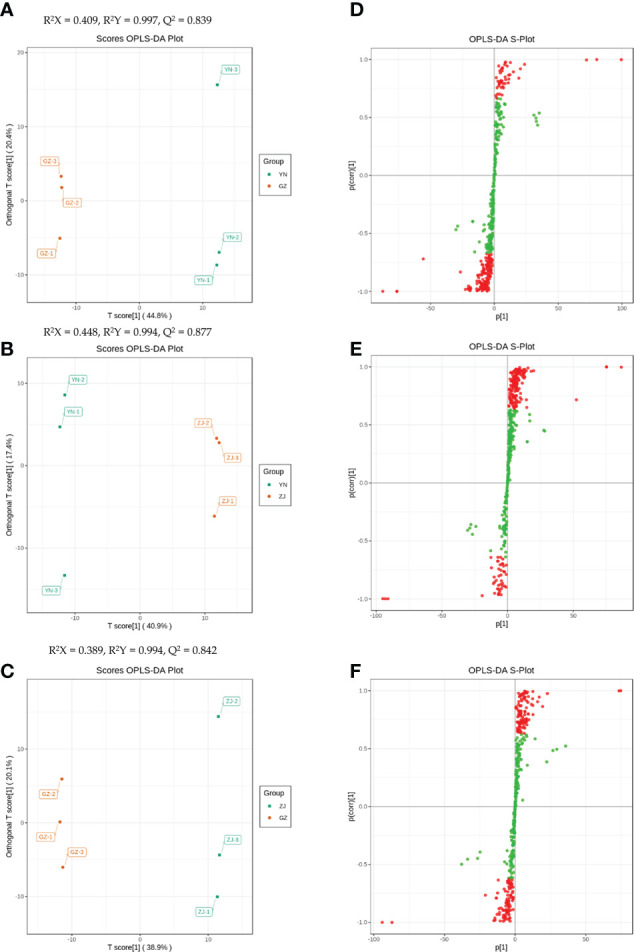
Orthogonal partial least squares-discriminant Analysis (OPLS – DA) scores. Scores of the OPLS – DA model with **(A)** YN vs GZ; **(B)** YN vs ZJ; **(C)** ZJ vs GZ. OPLS – DA s-plot model with **(D)** YN vs GZ; **(E)** YN vs ZJ; **(F)** ZJ vs GZ. R2Yscores and Q2values represent the interpretation rate of the model to the Y matrix and the prediction When Q2> 0.5, the model can be considered an effective model, And Q2> 0.9 is an excellent model.

Difference analysis was performed for the substances detected in all samples, and compounds with fold-change (FC) ≥ 2 or FC ≤ 0.5 and OPLS-DA VIP values ≥ 1 were defined as DAMs. In this study, the OPLS-DA model compared metabolite contents of the provenances in pairs to evaluate the differences between YN and ZJ (R^2^X = 0.409, R^2^Y = 0.997, Q^2^ = 0.839), YN and GZ (R^2^X = 0.448, R^2^Y = 0.994, Q^2^ = 0.877), and ZJ and GZ (R^2^X = 0.389, R^2^Y = 0.994, Q^2^ = 0.842) (**Figures 3A–C**). The Q^2^ values of all comparison groups exceeded 0.8, demonstrating that these models were stable. OPLS-DA score plots showed that the three provenances were well-separated in pairs, suggesting significant differences in metabolic phenotypes (**Figures 3A–F**).

### 3.6 Differential metabolite screening, functional annotation, and enrichment analysis among the three *D. officinale* provenances

**Figure 4:**
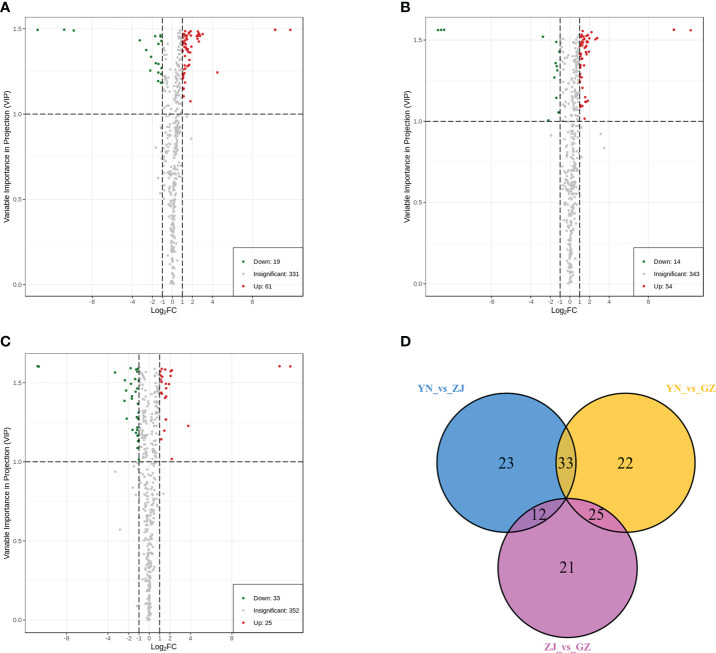
Volcano maps and venn diagram analysis of differential metabolites for three comparison groups (YN vs. GZ, YN vs. ZJ, ZJ vs. GZ). Differential metabolite analysis of *Dendrobium officinale* from different provenances. **(A–C)** Volcano maps of differential metabolites in different pairwise comparisons: **(A)** YN vs GZJ; **(B)** YN vs ZJ; **(C)** ZJ vs GZ. **(D)** Venn diagram shows the overlapping and unique metabolites amongst the comparison groups.

Based on fold change ≥ 2 or ≤ 0.5 and VIP ≥ 1, the number of up- and downregulated compounds resulting from pairwise comparison of tested species is shown in (**Figures 4A–C**) and (**Table 3**). The smallest number of differential metabolites was found in the ZJ vs. GZ group, with 58 (25 were upregulated and 33 were downregulated). Moreover, there were 80 significantly different metabolites between YN and GZ (61 upregulated, 19 downregulated) and 68 between YN and ZJ (54 upregulated, 14 downregulated). After taking the intersection of each comparison group in a Venn diagram (**Figure 4D**), no metabolites were shared among the comparison groups (YN vs. ZJ/GZ, ZJ vs. GZ). Moreover, there were 33, 12 and 25 differentially expressed metabolites between YN vs. ZJ and YN vs. GZ, YN vs. ZJ and ZJ vs. GZ, and YN vs. GZ and ZJ vs. GZ, respectively. These results showed that the metabolites that caused the differences between YN and ZJ, GZ were noticeably different. Among the differential metabolites, the contents of dihydropinosylvin methyl ether, quercetin-3-O-sophoroside-7-O-rhamnoside, quercetin-3-O-(4’’-O-glucosyl)rhamnoside, quercetin-3-O-glucoside-7-O-rhamnoside, orientin-7-O-glucoside and quercetin-7-O-rutinosider were relatively high in the three provenances, and the DAMs were relatively large. Noteworthy, phenolic acids (dihydropinosylvin methyl ether) in GZ provenance were 22.60-fold and 13.67-fold higher than in YN provenance and ZJ provenance, respectively, and phenolic acids in ZJ provenance were 1.65-fol higher than in YN provenance.

**Figure 5:**
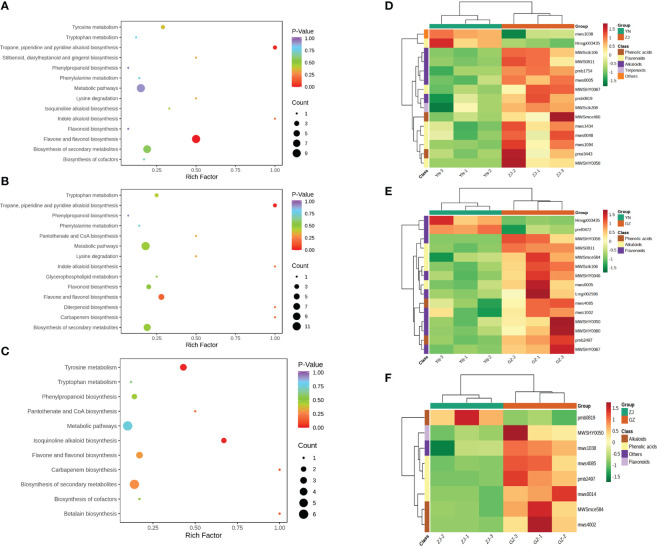
KEGG enrichment maps and heatmaps analysis of differential metabolites for three comparison groups (YN vs. GZ, YN vs. ZJ, ZJ vs. GZ). **(A–C)**, KEGG enrichment maps of differential metabolites in different pairwise comparisons: **(A)** YN vs. ZJ; **(B)** YN vs. GZ; **(C)** ZJ vs. GZ. D-F, Heatmaps of hierarchical cluster analysis (HCA): **(D)** YN vs ZJ; **(E)** YN vs GZ; **(F)** ZJ vs GZ. **(A–C)** The abscissa represents the enrichment factor of the pathway and the ordinate shows the names of pathways. The color of the dot represents the p-value, and the deeper the red of the dot, the stronger the enrichment effects. The size of points represents the number of metabolites enriched in the pathways. **(D–F)** The abscissa is used to display the names of samples, and the ordinate on the right is used to display the names of differential metabolites. The deeper the red color, the higher the content of the metabolites; the deeper the green color, the lower the content of the metabolites. The abscissa is the name of the sample and the ordinate is the differential metabolite. Different colors in the heat map represent the relative content of the differential metabolite. The value obtained after normalization reflects the relative content (red represents high content and green represents low content).

To explore the metabolite information in YN, ZJ and GZ provenances, we used the KEGG database to annotate and enrich the differential metabolites. In YN vs. ZJ, YN vs. GZ and ZJ vs. GZ groups, 16, 17 and 10 DAMs were annotated by KEGG and showed significant differences, respectively. The major pathways are presented in bubble plots (**Figures 5A–C**). Most noteworthy, in the YN vs. GZ comparison group, the DAMs were mainly enriched in “flavone and flavonol biosynthesis” and “tropane, piperidine and pyridine alkaloid biosynthesis” metabolic pathways (p-value < 0.05). In the YN vs. ZJ comparison group, the DAMs were mainly enriched in the “pyridine alkaloid biosynthesis” metabolic pathway (p-value < 0.05). In the ZJ vs. GZ comparison group, the DAMs were primarily enriched in “isoquinoline alkaloid biosynthesis” and “tyrosine metabolism” metabolic pathways (p-value < 0.05).

To more comprehensively and visually show the relationship between samples and screen marker metabolites, we performed hierarchical clustering of samples across groups using the expression levels of significant differential metabolites. Hierarchical cluster analysis (HCA) showed that the marker metabolites of *D. officinale* from the three provenances were mainly flavonoids, alkaloids and phenolic acids. The contents of l-tyramine (MWSmce584), sinapic acid (mws4085), 4-hydroxy-3-methoxyamygdaloic acid (pmb2497) and kaempferol-3-O-rutinoside (MWSHY0050) in GZ were significantly higher than those in YN and ZJ. Moreover, the contents of 2-phenylethylamine (MWSslk106), L-pipecolic acid (MWS0811), tryptamine (mws0005) and 5,7,3’,4’-tetrahydroxyflavone (MWSHY0058) in YN were significantly lower than those in GZ and ZJ (**Figures 5D–F**).

### 3.7 Correlation analysis of the secondary metabolites in *D. officinale* and their antioxidant activity

**Figure 6:**
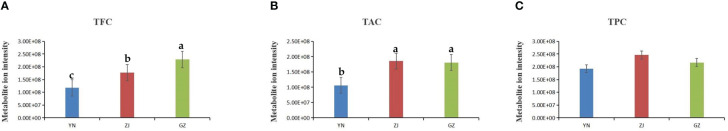
Comparison of the total ion intensity of various class of metabolites among stems from YN, ZJ and GZ. **(A)** TFC, total flavonoid content, **(B)** TAC, total alkaloid content, **(C)** TPC, total phenolic acid content. Different lowercase letters represent significant differences.

We performed correlation analysis for all flavonoids, phenolic acids and alkaloids detected in the three materials because these compounds are the major antioxidants in plants (Zhang et al., 2017) and account for the largest proportion of *D. officinale* constituents. The total flavonoid content (TFC) from GZ provenance *D. officinale* fractions was significantly higher than that from ZJ provenance, and TFC from ZJ provenance *D. officinale* fractions was significantly higher than that from YN provenance. Measured total alkaloid content (TAC) revealed that extracts from GZ and ZJ provenances had significantly higher levels than the *D. officinale* stem extract from YN provenance. In contrast, total phenolic acid content (TPC) in extracts had no significant difference among the three provenances (**Figure 6**). The results showed that TFC may be closely related to the antioxidant activity of the three species of *D. officinale*.

**Figure 7:**
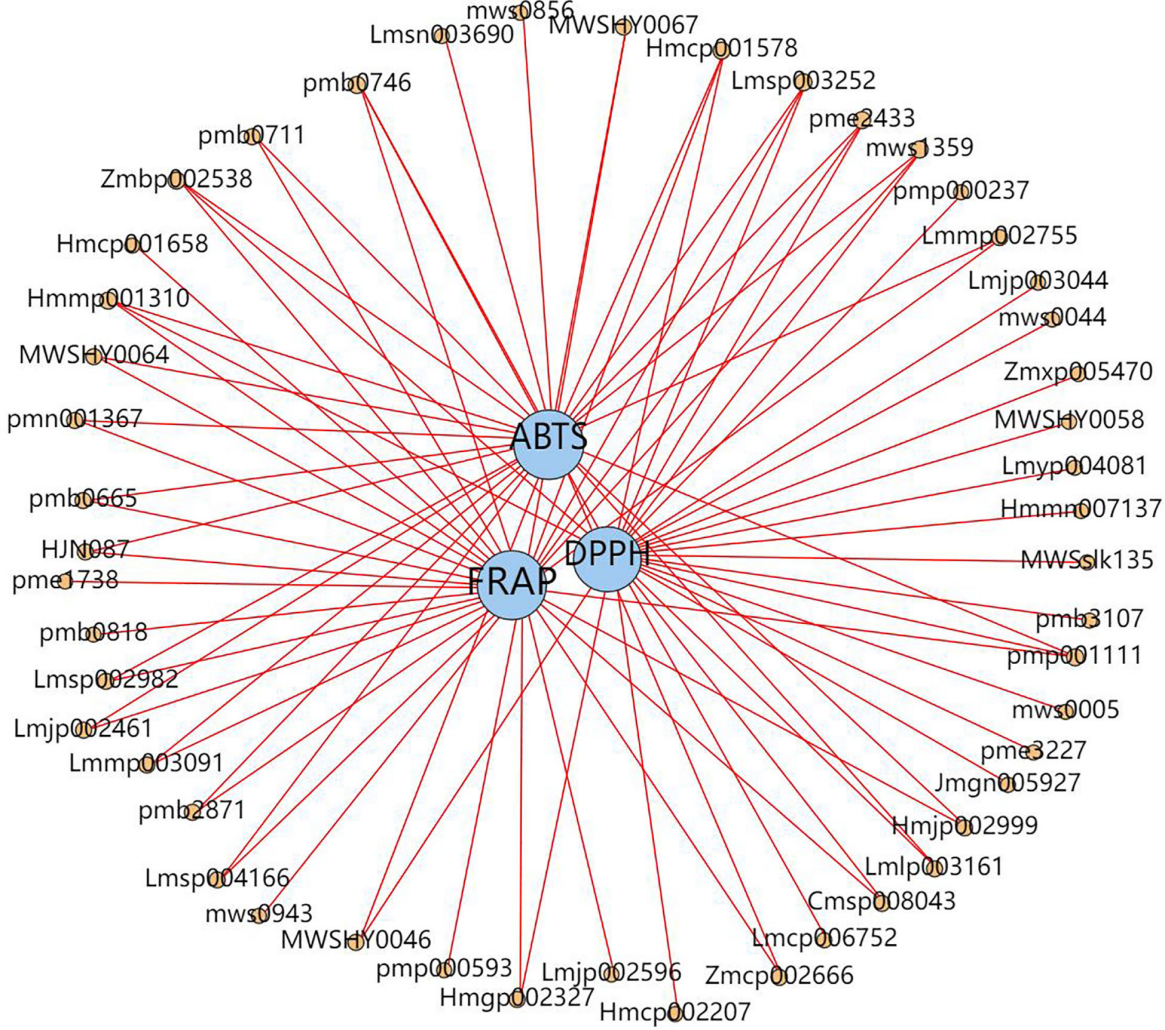
Network diagram between antioxidant capacity and metabolites. Blue circles indicate different antioxidant capacity (DPPH, 2,2-diphenyl-1-picrylhydrazyl radical scavenging ability; ABTS, 2,2’-Azinobis- (3-ethylbenzthiazoline-6-sulphonate; FRAP, ferric reducing antioxidant power.), orange circles indicate different metabolites (The number above the circle indicates the component ID of Metware), and the line connecting the two circles represents the correlation (r≥0.8, p < 0.0092).

We examined the correlation (Spearman correlation coefficient) between all the different metabolites detected and antioxidant activities (ABTS, DPPH, FRAP) to gain further insight into the ingredients of antioxidants present in *D. officinale* and identified 48 metabolites that displayed a significant positive correlation (r ≥ 0.8, p < 0.0092) with antioxidant activity. Here, 30 flavonoids, 9 alkaloids, 4 phenolic acids, 2 stilbenes, 1 sesquiterpenoid, 1 coumarin and 1 other substance displayed a significant positive correlation with at least one antioxidant activity, suggesting that in addition to flavonoids, alkaloids and phenolic acids, some stilbenes and other substances in *D. officinale* may also be important antioxidants (**Figure 7**). Moreover, flavonols (quercetin-3-O-neohesperidoside, quercetin-3-O-(4’’-O-glucosyl)rhamnoside, quercetin-7-O-rutinoside, quercetin-3-O-rutinoside (Rutin) and quercetin-3-O-sophoroside-7-O-rhamnoside) levels in GZ extracts were 3.92- and 1.84-fold, 4.65- and 1.93-fold, 4.55- and 1.91-fold, 5.71- and 1.83-fold, 6.64- and 4.56-fold higher than in YN and ZJ extracts, respectively. alkaloids (1-methoxy-indole-3-acetamide, 3-amino-2-naphthoic acid and 3-Indoleacrylic acid) levels in GZ extracts were 1.40- and 1.13-fold, 1.48- and 1.16-fold, 1.50- and 1.20-fold higher than in YN and ZJ extracts, respectively. phenolic acids (1-O-Gentisoyl-β-D-glucoside, glucosyringic acid and dihydropinosylvin methyl ether) levels in GZ extracts were 1.59- and 1.42-fold, 1.94- and 1.76-fold, 71.06- and 13.67-fold higher than in YN and ZJ extracts, respectively (**Table S1**). Among them, the correlation coefficient between flavonols (quercetin-3-O-neohesperidoside, quercetin-3-O-(4’’-O-glucosyl)rhamnoside, quercetin-7-O-rutinoside, quercetin-3-O-rutinoside (Rutin) and quercetin-3-O-sophoroside-7-O-rhamnoside) and antioxidant index was 0.867, 0.867, 0.867, 0.900 and 0.833 for ABTS, respectively. The correlation coefficients of alkaloids (1-methoxy-indole-3-acetamide, 3-amino-2-naphthoic acid and 3-Indoleacrylic acid) were 0.954, 0.867 and 0.950 for FRAP, respectively. The correlation coefficients between 1-O-Gentisoyl-β-D-glucoside and the antioxidant indexes were 0.883 for ABTS; The correlation coefficients between glucosyringic acid and the antioxidant indexes were 0.812 for DPPH; The correlation coefficients between dihydropinosylvin methyl ether and the antioxidant indexes were 0.837 for DPPH (**Table S2**). According to the multiple and correlation coefficient, we infer thatwe infer that content of quercetin-3-O-sophoroside-7-O-rhamnoside and dihydropinosylvin methyl ether might be the underlying causes of the differences in antioxidant capacity and pharmacological effects of YN, ZJ, and GZ.

## 4 Discussion

### 4.1 The secondary metabolites of *D. officinale* from different provenances were significantly different

The provenance is the regional adaptive varieties derived from different natural distribution areas or formed from geographical isolation through artificial cultivation and domestication. There may be great differences in the growth, development and quality of different provenances, which is the basis for the selection and cultivation of excellent varieties.

Lan et al., indicated that a total of 442 metabolites were identified in three different origins from Guangxi Province (GP), Guangdong Province (RS) and Zhejiang Province (ZJ), and were divided into 16 major categories, among including 244 differential metabolites Lan et al. (2022). In our study, 411 metabolites were identified including 8 categories, among 136 differential metabolites were identified in the three comparison groups from YN, ZJ, and GZ. Compared with previous studies, the metabolites, categories, differential metabolites have decreased, it may be that our three provenance materials are cultivated in the same place, which excludes the influence of growth environment on metabolites. By comparing the species and content of different metabolites between YN, ZJ provenances and GZ native provenances, the difference is mainly reflected in flavonoids, alkaloids and phenolic acid metabolites. Besides, the content of quercetin-3-O-rutinoside noticeably different, while the *in vivo* radiation protection effect of quercetin-3-o-rutinoside has been evaluated (Bansal et al., 2012) and may represent a quality evaluation index. Similarly, the content of l-pipecolic acid significantly different, while previous study indicated that Pipecolic acid (Pip) biosynthesis and hydroxylated modification pathways play a critical role in drought tolerance through the antioxidant system in tomato (Wang et al., 2021). Thus, these compound may also be used as a quality evaluation index. In this study, the content of l-tyramine was observed to be noticeably different, and previous study showed that l-tyramine also serves as a precursor of numerous specialized metabolites that have diverse physiological roles as antioxidants and defense compounds in plant (Schenck and Maeda, 2018). Furthermore, vicenin-2 was detected in *D. officinale* from the three different provenances, and the abundance of this compound is relatively large, which is consistent with a previous study (Xu et al., 2013). Besides, the *D. officinale* also contains a variety of flavonoid carbonosides with apigenin as an aglycone (Chen et al., 2021). Thus, these metabolites can be used to evaluate the quality of *D. officinale*.

The growing environment of plants affects the changes in metabolites in plants. Important factors affecting the accumulation of secondary metabolites in plants include different altitude, temperature, and Light. It has been observed that the production of polysaccharides *D. officinale* decreases gradually, with the increase in altitude and decrease in temperature (He et al., 2022). Light-treatment affected the accumulation of primary and secondary metabolites in *D. officinale*, especially the biosynthesis of flavonoids, which the components of flavonoid glycosides were significantly upregulated (Jia et al., 2022). Zuo et al. (2020) conducted a comparative analysis of the metabolomics of *D. officinale* under different cultivation substrates, and they found that only three shared metabolites differed significantly among the different comparisons. Lan et al. (2022) conducted a comparative analysis of the metabolomics of *D. officinale* under different region, and found that were 22 significantly different metabolites shared among the three comparison groups. Nevertheless, in our study, there weren’t significantly different metabolites shared among the three comparison groups. This difference indicates that provenance is the main factor affecting the differential metabolites of *D. officinale*, while the growing environment has more lesser. In summary, YN and ZJ provenances were introduced to Guizhou Province for cultivation, which the homogeneity of the cultivation environment in the later period may lead to the decrease of some differential metabolites.

### 4.2 Relationship between composition and content of secondary metabolites of *D. officinale* and antioxidant capacity

We identified 48 different metabolites that displayed a significant positive correlation with antioxidant activity in *D. officinale*. Among them, the quantity and content of flavonoids were the most abundant. Flavonoids are important secondary metabolites, and flavonoids in medicinal plants have been shown to have various biological and pharmacological activities such as anti-diabetic, anti-cancer and antioxidant (Mo et al., 1992; Wang et al., 2006; Wang et al., 2019). Huang et al. (2022) showed that the contents of polyphenols and flavonoids in alcohol extracts were positively correlated with the *in vitro* antioxidant capacity of *D. officinale*. While, in this study, we found that flavonoid compounds accounted for the highest proportion of total metabolites, meanwhile the total flavonoids content and total alkaloids content had significant positive correlation with antioxidant activity, which is consistented with previous studies. However, the total phenolic acid content hadn’t significant positive correlation with antioxidant activity, inconsistent with previous studies (Huang et al., 2022). This indicates that phenolic acids have little effect on the antioxidant activity of the three provenances. Flavonols (quercetin-3-O-glucoside-7-O-rhamnoside, quercetin-3-O-neohesperidoside, quercetin-3-O-(4’’-O-glucosyl)rhamnoside, quercetin-7-O-rutinoside and rutin) were significantly and positively correlated with the antioxidant capacity of *D. officinale*, and the content of metabolites was relatively high. This observation is consistent with previous reports that flavonols are important antioxidants (Jonathon and Bozzo, 2015). Equally, the antioxidant activity of stems from *D. officinale* showed a positive correlation with alkaloids (1-methoxy-indole-3-acetamide, 3-amino-2-naphthoic acid and 3-Indoleacrylic acid), which is consistent with a previous study [Tang et al., 2021]. In addition, previous studies have shown that many phenolic acid compounds in plants also have antioxidant activity, we found that phenolic acids (1-O-Gentisoyl-β-D-glucoside, glucosyringic acid and dihydropinosylvin methyl ether) were significantly and positively correlated with the antioxidant capacity of *D. officinale*, which is consistent with previous studies (Pradeep and Sreerama, 2018). Therefore, these metabolites can be used as quality evaluation indicators for screening high-quality provenances of *D. officinale*, which has certain reference significance for industrial planting and promotion of *D. officinale*. We found that the contents of quercetin-3-O-sophoroside-7-O-rhamnoside and dihydropinosylvin methyl ether in three different provenances were the most different. Whereas, in the previous studies quercetin-3-O-sophoroside-7-O-rhamnoside and dihydropinosylvin methyl ether has not been reported in *D. officinale*. It can be further studied on the separation, purification and function of these landmark metabolites.

## 5 Conclusions

The secondary metabolic composition and antioxidant capacity in *D. officinale* from three different provenances (GZ, ZJ, YN), were systematically studied for the first time. In total, 411 metabolites were identified including 8 categories such as flavonoids and phenolic acids, 136 of which were differential metabolites. These differentially accumulated metabolites (DAMs) were enriched in secondary metabolic pathways such as “flavone and flavonol biosynthesis”, “tropane, piperidine and pyridine alkaloid biosynthesis”, “isoquinoline alkaloid biosynthesis” and “tryptophan metabolism”. The metabolites among the three *D. officinale* showed significant differences, mainly differences in accumulated quantity. GZ has the highest TFC and the highest antioxidant activity, followed by ZJ. Correlation analysis identified that 48 differential metabolites showed a significant positive correlation with antioxidant capacity, and flavonoids were the main factors affecting the different antioxidant activities. Quercetin-3-O-sophoroside-7-O-rhamnoside and dihydropinosylvin methyl ether might be the underlying causes of the differences in antioxidant capacity of three *D. officinale*. This study provided reference for the breeding, quality control and product development of *D. officinale*.

## Data availability statement

The original contributions presented in the study are included in the article/**Supplementary Material**. Further inquiries can be directed to the corresponding author.

## Author contributions

Conception and design: QN and LL. Analysis and interpretation: ZL. Data collection: ZL, LL, MH, CL, JY and FD. Provide the materials: YS and YM. Writing the article: ZL and QN. Final approval of the article: QN. All authors have read and agreed to the published version of the manuscript. All authors contributed to the article and approved the submitted version.
